# Supplementary material for: Automated phenol-chloroform extraction of high molecular weight genomic DNA for use in long-read single-molecule sequencing
Source: F1000Res. 2022 Feb 28;11:240. [Version 1] doi: 10.12688/f1000research.109251.1 (PMC8931447; doi:10.12688/f1000research.109251.1)
Supplement: Peer review history

# Peer review: Reviewer response for version 1

**DOI:** 10.5256/f1000research.120730.r125732

**Published:** 2022-03-17

**Authors:** Shan Wei

**Affiliations:** 1 Columbia University Irving Medical Center, New York City, NY, USA

The manuscript describes the development of a Phenol-Chloroform high molecular weight (HMW) DNA extraction protocol using a dual-arm anthropomorphic robot and the application on biological samples including algae, bacteria, insects, and human cell lines.

 Automation of Phenol-Chloroform DNA extraction protocol is technically challenging. It requires handling the aqueous phase in the presence of organic solvent precisely. As addressed in the manuscript, Phenol-Chloroform DNA extraction is time-consuming and involves exposure to toxic organic solvents. It has a moderate to high gDNA extraction yield. However, Phenol-Chloroform DNA extraction recovers HMW gDNA due to minimum mechanical shearing in the protocol. The emerging long-read sequencing techniques such as Nanopore or PacBio are sharp tools in building genome assemblies and studying structural rearrangements. High-quality HMW DNA is fundamental to long-read sequencing.

 The manuscript is well organized and provided sufficient details. The protocol was demonstrated on different types of specimens. The extracted gDNA was used to prepare libraries for nanopore long-read sequencing, which was described in another study from the same research group. The problem-solving section along with the successful development of the automation protocol is very helpful to the scientific community. Overall, this is a well-written manuscript with solid data support. There are two minor comments/suggestions for the authors:

1. Using this automated protocol, what’s the range of gDNA concentration and OD (Optical Density) 260/230 extracted from samples used in this study? 
2. The pore occupancy and sequence yield from the nanopore sequencing runs are relatively low, indicating optimizations are needed for the nanopore library preparation and loading for future development.

Is the work clearly and accurately presented and does it cite the current literature?

Yes

If applicable, is the statistical analysis and its interpretation appropriate?

Not applicable

Are all the source data underlying the results available to ensure full reproducibility?

Yes

Is the study design appropriate and is the work technically sound?

Yes

Are the conclusions drawn adequately supported by the results?

Yes

Are sufficient details of methods and analysis provided to allow replication by others?

Yes

Reviewer Expertise:

Genetics, Molecular Biology, Bioinformatics, Long-read Sequencing

I confirm that I have read this submission and believe that I have an appropriate level of expertise to confirm that it is of an acceptable scientific standard.

# Peer review: Reviewer response for version 1

**DOI:** 10.5256/f1000research.120730.r125733

**Published:** 2022-03-08

**Authors:** Genki N. Kanda

**Affiliations:** 1 RIKEN Center for Biosystems Dynamics Research, Osaka, Japan

This paper demonstrates the implementation of the phenol-chloroform DNA extraction procedure, one of the most commonly used experimental techniques in molecular biology, on a dual-armed humanoid robot. The scope of the paper is quite simple, and it clearly and concisely describes not only the results of the implementation, but also the limitations and solutions during development. In the field of laboratory automation, it is greatly useful to share the difficulties encountered during development, and the authors' description should be of sufficient help to the readers.  

 In the introduction, the authors state that *"Automating the procedure was expected to enhance reproducibility, increase throughput, and reduce the possibility of DNA shearing into shorter fragments by minimizing excessive handling."* It would be more helpful to the readers if the conclusions about reproducibility, throughput, and quality were described in the discussion.

Is the work clearly and accurately presented and does it cite the current literature?

Yes

If applicable, is the statistical analysis and its interpretation appropriate?

Not applicable

Are all the source data underlying the results available to ensure full reproducibility?

Yes

Is the study design appropriate and is the work technically sound?

Yes

Are the conclusions drawn adequately supported by the results?

Yes

Are sufficient details of methods and analysis provided to allow replication by others?

Yes

Reviewer Expertise:

Laboratory Automation, Molecular and Cellular Biology

I confirm that I have read this submission and believe that I have an appropriate level of expertise to confirm that it is of an acceptable scientific standard.
